# Epidemiological Review of Scorpion Envenomation in Iran 

**Published:** 2014

**Authors:** Amir Jalali, Fakher Rahim

**Affiliations:** a Department of Pharmacology and Toxicology, School of Pharmacy and Toxicology Research Center, Ahvaz Jundishapur University of Medical Sciences, Ahvaz, Iran.; b Toxicology Research Center, Ahvaz Jundishapur University of Medical Sciences, Ahvaz, Iran.

**Keywords:** Scorpion, Iran, Geographical distribution, Envenoming, Fauna

## Abstract

This epidemiological review was carried out to display the magnitude and the geographic distribution of scorpion envenomation in Iran with focus on the southwestern region of Iran, particularly. The Iranian recognized scorpions belonging to two families, including Buthidae and Scorpionidae. Buthidae family consists of 14 genuses, 26 species, and 18 sub-species, while Scorpionidae family has three genuses and four species. The lack of basic knowledge, including the geographical distribution, clinical manifestations, and specific treatments related to scorpiofauna justifies such multidisciplinary studies. The venom of two endemic Iranian scorpions, including *Hemiscorpius lepturus *(*H. lepturus*) and *Odonthubuthus doriae *(*O.doriae*) have considered as an effective source of new neurotoxin peptides for the further development of physio-pharmacological probes and designing the clinical trials. Such epidemiological information may improve the determinants of Iranian scorpion stings in order to plan and implement effective public health intervention.

## Introduction

Scorpions spread around the world widely in different places, except in the South Pole. However some of the ocean islands such as New Zealand, United Kingdom don’t have any native scorpions but five colonies of scorpions (Euscorpius flavicaudis) have settled themselves in southern England. The accumulation of scorpions is high in the half-warm areas (bearing of 23-38 geographical width). They would get less in varieties as we go towards the poles and smaller (in size) as we go towards the tropical areas (1). 

Iran is thought to have many remarkable venomous animals’ fauna such as scorpion. Different fatal species such as *Andrectonus *genus are found in throughout the semi-arid and arid regions in the Iranian neighbor’s countries except to the north countries. 

Stings by venomous animals are a concern to health authorities in many Middle-east countries because of the severity, extent, and a wide range of clinical effects. Although various scorpions are exist, majority of stings result in cardiotoxicity, neurotoxicity and respiratory dysfunctions (2, 3). Despite the great number of Iranian scorpion species, only a few are studied or even considered as being truly dangerous. This fauna, whether large or small have venoms with the user in different medial area. All investigated venom contains several toxins. The examined toxins have being used as insecticide, pharmacological and physiological probes, protein engineering scaffolds and even to treat cancer patients. After all, if we couldn’t present this fauna for all and get results back, these significant fauna wouldn’t be of much use. For presenting this information, a common method used is a review which shows the new data. 

Iranian southern areas have high scorpion stings incidence with all dangerous and medically important scorpion’s distribution. So this review concentrates primarily on the geographical distribution of the scorpiofauna in these parts. Accordingly, two areas of Omani Gulf (Provinces at the side of Persian Gulf and Oman Sea) and Southern Zagros Mountain are considered for our study and research. Therefore, the main objective was to find the living characterizations, different available species, the frequency and community which help us to hunt different groups of scorpion species and acquire their venoms, at the side of looking at their geographical distribution. 

However, there is a distinct lack of knowledge of the venom composition of the most scorpions in Iran; this study also is the work of researches of isolated neurotoxins from two Iranian scorpions *Hemiscorpius lepturus *(*H. lepturus*) and *Odonthubuthus doriae (O.doriae). *This review brings up the distinction between Iranian and worldwide scorpion species, characterization pharmacological activities and the main clinical manifestations following stings. The main objective of this study is to review the results of the various published or available epidemiological, electrophysiological, clinical or even pharmacological studies leading up to this distinction. 

*Iranian scorpion’s fauna*

**Figure 1:**
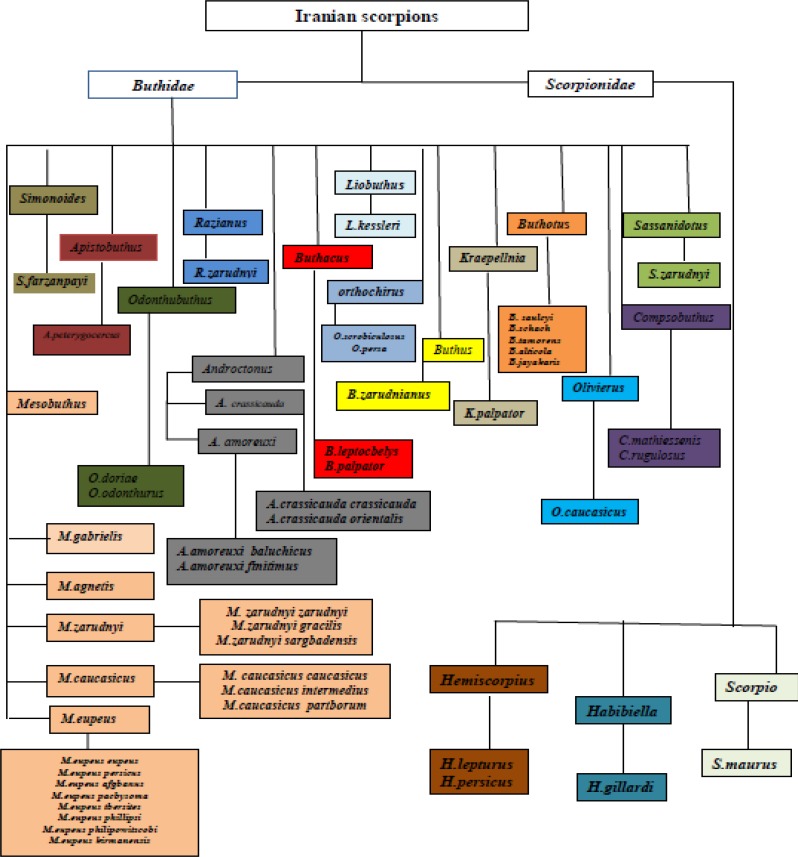
Iranian scorpion’s fauna

The Iranian scorpion’s fauna has two families, which consists of over 30 named species and 18 subspecies from 17 genuses. The families are Buthidae and scorpionidae. So far, the identified genus’s are *Scorpio, Habibiella, Hemiscorpius *(from Scorpionidae family), *Olivierus, Kraepellnia, Compsobuthus, Sassanidotus, Buthus, Buthacus, Liobuthus, Buthotus, Andrectonus, Odonthubuthus, Orthochirus, Mesobuthus, Apistobuthus and Simonoides*. Of these, at least 7 species including *H. lepturus, O. doriae, Buthotus salcyi, Buthotus sach*, *Andrectonus crassicauda, Mesobuthous eupous *and *Apisthobuthus petrigus *are medically important, and implicated in envenoming of humans (4) (Figure 1). 

The varieties of scorpions in Iran are outstanding in the context of the country’s environmental and geographical situation. However, our information is so poor and limited about the living facts and the exact geographical publication of Iranian scorpions. 

*Medically important Iranian scorpions: Toxinology and distribution*

Scorpion venom has potent disulfide-rich peptide that selectively target specific types of ion channels and receptors. This affinity made these neurotoxins important pharmacological tools and therapeutic potential (for more reviews of scorpion toxins see Possani *et al*., 1999) (5, 6). 

**Figure 2:**
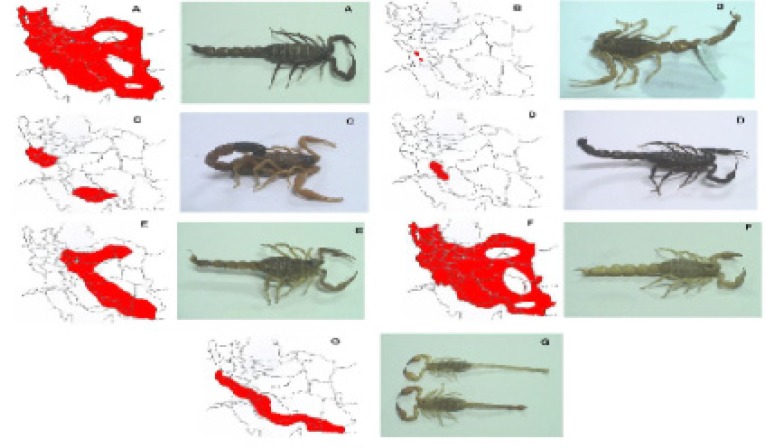
Geographical distributions of different scorpions across the Iran; A, *Andrectonus crassicauda*; B, *Apisthobuthus petrigus; C, Buthotus (Hotenttota) salcyi; D, Buthotus schach; E, O. doriae; F, Mesobuthus eupeus; G, H. lepturus.*

**Figure 3:**
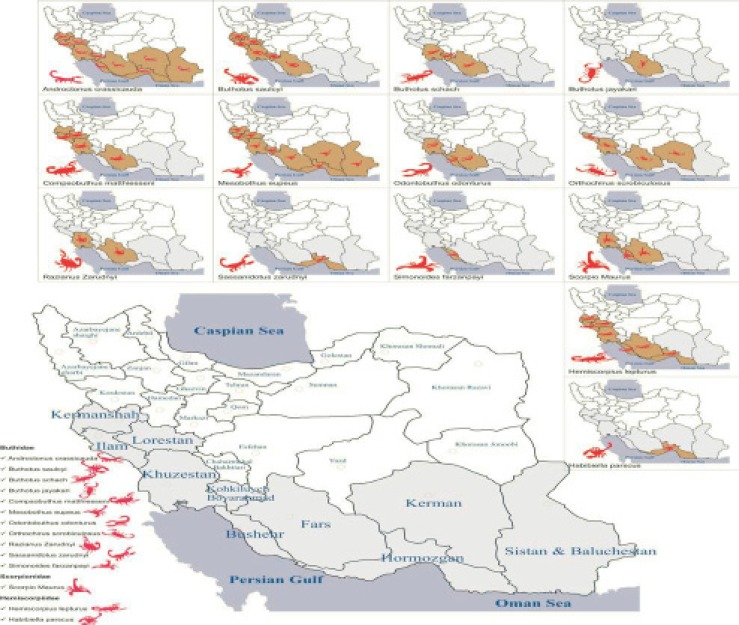
South and Southwest geographical distribution of Iranian scorpions Iranian scorpion envenomation.

Iranian scorpions are widely distributed (see distribution map of the medically important Iranian scorpions) (Figures 2 and 3). However, there is an obvious lack of knowledge of the venom composition of the most scorpions. Some of the poor information about Iranian scorpions is because of inadequate descriptions of species and the lack of illustrations, particularly of rare type of scorpions. The most distinguished exceptions almost are two scorpions, Iranian yellow scorpion (*O.doriae*) and Iranian scorpion (*H.lepturus*). 

Up to date, six neurotoxins are isolated from their venom and have become the focus for other studies. These toxins were OD1, an α-like toxin active on voltage-gated sodium channels (8), OdK1, a selective inhibitor of K_v_1.2 channels (9); OdK2, a K_v_1.3 channel-selective toxin from the venom of the Iranian yellow scorpion *O. doriae *(10), Hemicalcin, a selective neurotoxin against ryanodin-sensitive Ca^2+^ channels (11), Hemitoxin, a selective inhibitor of K_v_1.1, K_v_1.2 and K_v_1.3 channels (12) and Heminecrolysin, a hemolytic dermonecrotic toxin with strong hemolytic capacity from the venom of the Iranian scorpion *H. lepturus *( 13). 

The available polyvalent antivenom is prepared by by the Razi Vaccine and Serum Production and Research Institute through hyper immunizing healthy horses against the 6 medically important scorpion species *Odontobuthus doriae, Mesobuthus eupeus, Androctonus crassicauda, Buthotus (Hottentota) Saulcyi, Buthotus sach *and *H. lepturus *(7). Next freeze-drying preparation, the scorpion venom can be stored with sufficient toxicity and enzyme activity at room temperature for a long period of time. The mean yielded from milking of the most dangerous Iranian scorpions is variable and presented in Table 1. The dried venom has a ranging from 0.13 (*H. lepturus*) to 1.6 mg/ scorpion (*Buthotus (Hottentotta) saulcyi*). Much higher dried venoms are obtained from larger species *Buthotus (Hottentotta) saulcyi *and *Androctonus crassicauda*. Low amount is obtained from smaller species *H. lepturtus, Mesobuthus eupeus *and *Scorpio maurus*, although the toxicity of venom from *H. lepturus *is usually significant and showed more severe clinical manifestations. Table 1 provides a vastly different LD_50_ of the most dangerous Iranian scorpions. *Androctonus crassicauda *(0.08/iv method) and *Scorpio maurus *(9.37/iv method*) *have the highest and lowest potent venom, respectively. The venom of *H. lepturus *has much less potent venom. The precise assessment of LD_50_ is an important step for evaluation of antivenom potency and the optimization of its efficacy. The available antivenom (5-mL ampoules) is a pepsin-digested refined product. As showed in Table 1, each 1 ampoule is able to neutralize the whole venom of the species and rescuing victims from severe envenomation. Therefore, it is likely that administration of this polyvalent antivenom is a therapeutic measure to circumvent the medical important Iranian scorpions envenoming. 

**Table 1:** variation in yield and lethality of venom from some Iranian scorpions and potencies of available polyvalent scorpion antivenom

**Species **	**Dried venom mg/scorpion**	**LD**_50_ **/ method**	**Antivenom neutralized mg/ml**
*Androctonus crassicauda *	1.0 ± 0.4	0.08-0.5 SC or iv	0.04 ± 0.03
*Buthotus (Hottentotta) saulcyi *	1.6 ± 0.4	1.01 iv	0.38 ± 0.05
*Hemiscorpius lepturtus *	0.13 ± 0.06	5.81 iv	0.92 ± 0.24
*Mesobuthus eupeus *	0.3 ± 0.2	1.45 iv	0.36 ± 0.19
*Odonthubuthus doriae *	0.6 ± 0.1	0.19 iv	0.17 ± 0.09
*Scorpio maurus *	0.2 ± 0.1	9.37 iv	0.8 ± 0.14

The LD_50_ expressed in mg of venom per Kg of mouse; Method: iv: intravenous injection; SC=subcutaneous injection

The dried venom and antivenom neutralized potency are expressed in mg ± SD

*Epidemiology of scorpion envenomation in southwestern region of Iran: areas of potential risk*

This geographic area includes two different environmental characteristics with two different geographical locations. It consists of the regions at the side of the Oman Sea and the Persian Gulf. These particular regions have less raining and are warm. Their winter is temperate and has the semi-equator vegetation coverings. The southern Zagros which is part of Zagros Mountain is from the south of Bakhtaran province and Elam Province to Kerman province. These regions are mostly covered by the herbaceous regions and there are bushy acorn jungles. There is a less moisture in the air and these areas have warm summers and cold winters. From these districts, which are covered by ten provinces, 40 regions (from each four regions) and 3-6 areas from each region according to their geographical conditions have contain scorpion (Figures 2 and 3). Scorpion sting has seen 8 months from the start of each year, which is the best time of scorpion hunting. In the remained month of year, their shapes, the final reorganizations, their adding up and analysis have been carried out. There have been enough samples and scorpions of each region with their life parameters, to be taken as the research values. Afterwards, all the samples have been transferred to the labs and after achieving their figures and, they were added up and analyzed in the labs.

According to report were received, it is known that Iran have one of the highest ranks in terms of scorpion bites over the world (existing data from Ministry of Health of Islamic Republic of Iran). The exact incidence of scorpion sting and death resulting from scorpion sting in Iran is unknown due to paucity of data from poisoning and envenomation cases in most regions. Nevertheless, in south west province, Khuzestan there are more than 50000 calls each year for information about scorpion stings to the Health Center of this province. In this investigation identified that after respiratory, infectious and digestive diseases, scorpions sting allocate the greatest number of deaths in the humid and oil-rich province, Khuzestan. In these province, dangerous scorpion for example *H.lepturus *and to a lesser extent *Anderoctunus crassicauda *species estimated cause of 100% death cases in each year. Although most cases of scorpion stings such as *Mesobuthus eupeous *result in minimal, if any, significant clinical consequences, the medically important scorpion stings normally produce either serious systemic clinical envenomation syndromes or significant local reaction particularly with *H.lepturus *(14). The extent of this problem has been addressed by Pipelzadeh and his colleagues where epidemiological and clinical scorpionism particularly severity and renal effects by the most identified hazardous scorpion of Iran (4), *H.lepturus *have reported (15). In following the envenoming characteristics, clinical manifestations, venom components, taxonomy and distribution of all the medically important Iranian scorpions will be discussed more. 

*Odontobuthus doriae: Toxinology and envenomation*

Arguably, the most wide-spread scorpions in Iran are *Andrectonus crassicauda, Mesobuthus eupeus a*nd the yellow Iranian scorpion *O.doriae *that particularly can be found in central and southern parts of Iran (Figure 2). Within the most dangerous scorpions in Iran, *Odonthubuthus*, from the Buthidae family and *Buthus *genus is one of them. *Buthus *is a genus that has two species *doriae *and *odonturus *(7). 

Despite highly distribution, little is known about the true incidence of envenomation due to a lack of organized data collection in most regions. Indeed, clinically significant the medically important Iranian scorpions envenoming requiring available antivenom therapy. The *O.doriae *sting regarded as potentially fatal with the most recent death attributed to scorpion stings occurring in central parts of Iran (according to existing reports and available information in Ministry of Health). Although the severity of envenomation by *O.doriae *is not as much as *H. lepturus *envenoming, sting of this scorpion may result in a variety of manifestations ranging from local pain, inflammation and necrosis to muscle paralysis and even hematuria (8). Although the frequency of bites by this scorpion is high, there are no studies that clearly describe the clinical effects of *O.doriae*. However, *O. doriae *sting may cause experimentally paralysis on sciatic nerve (16), anti-proliferative and apoptotic effects and reduces S-phase population in human breast cancer cells (17) and DNA synthesis inhibition (18). 

Specifically in Iran, clear clinical studies of the effects of the scorpion stings and effectiveness of antivenoms have been mainly carried out for two known scorpion *H.lepturus and A.crassicauda *(14, 19, 15). 

*O.doriae *sting may vary in severity depending on the age of victims, the season, and the amount of venom injected but to be similar regardless of the huge geographic distribution of this scorpion. Most commonly manifestation local pain and extended edema are noted at the site. Other manifestations may include sweating, vomiting, delirium and anxiety (20). Tremor and muscle fasciculation may be seen throughout the sting limb, in both limbs or in the contra-lateral limb only (21). The main feature of this scorpion, however, is pain (20, 22). When severe envenomation occurs, death may results but typically only in young children’s (8). Available records showed that the majority of stings cause clinical effects with severe pain like *A.crassicauda *sting. 

The mechanism of action of the majority of Iranian scorpion venom except for *H.lepturus *and *O.doriae *is difficult to discuss. Because a few toxins have been isolated and characterized in venom of *H. lepturus *with more specifically in the venom of O.doriae. 

More recently, the venom toxicity on neuromuscular transmission using extra and intracellular recording techniques was assayed (16, 21). The neurotoxic effects of *O.doriae *venom are likely to be due to peptide toxins that affect ion channels such as sodium and potassium channels in nerve-muscle preparations. The effects of *O.doriae *on reduction of potassium current are mostly due to toxins such as OdK1 which facilitate the release of acetylcholine (9, 23). Such effects could lead to initial enhancement of transmitter release and clinical tremors manifestation in envenoming (21). The overall action of *O.doriae *venom is likely to cause both presynaptic activities at low concentration of venom, whilst with high concentrations of venom the pharmacology would suggest a more myotoxic mechanism of action (21). 

Original studies of *O.doriae *venom were undertaken by Jalali, Abdel-motalleb that isolated, complete sequencing and study pharmacologic effects of two neurotoxins OD1 and OdK2 responsible for *O.doriae *envenomation (8-9). The effects of OD1 on three cloned VGSCs (Nav1.2, Nav1.5 and Para/TipE) are compared to what is known for other α-like toxins, electrophysiologically tested on cloned VGSCs. The main target of OD1, as α-like toxin, is evidently the insect voltage–gated sodium channel (VGSC), para/TipE, of which the inactivation process is completely inhibited. OD1 acts on VGSCs of both mammals and insect is in concordance with the reports that *O.doriae *venom has toxicity against insect larva also. This toxin seemed to be more active on insect than on mammals channels (21). 

However, the use of antivenin has been further challenged because it is potentially hazardous when allergic complications occur, but it is commonly believed that available antivenom therapy is the most effective treatment for the symptoms and signs of scorpionism (24). Available data in Razi Vaccine and Serum Production institute showed that antivenom administration is sufficient therapy in most of Iranian scorpion stings including *O.doriae *(Table 1). However death occurring after delay antivenom administration in severe envenomation may result but typically only in children or the very elderly. In Iran, a high percentage of *O.doriae *stings cause severe systemic effects such that antivenom is warranted in more than 90% of these cases (existing data). 

*Black scorpion (Andrectonus crassicauda): *Taxonomy, envenomation and distribution 

The size of this scorpion becomes about 10cm as an adult and is black/dark brown. They are normally long and reach 13 cm as an adult. This specious were hunted from the lowest sites of the region around the Persian gulf and Oman sea, from the height of 5m to 2000m from the sea level. Therefore, they are mostly spread in the southern part of Iran, horizontally. 

This scorpion is widespread in all part of Iran and is endemic to most Mediterranean countries and Africa (25). In particular, collected samples from the central Iranian desert Kavir have been brown or dark brown. Metasomal segments are reddish brown or brown with black pigment on keels. Its length reach to 10cm. *A. crassicauda *needs special attention because it lives close to human activities. Two subspecies of this species, *A. crassicauda crassicauda *and *A.crassicauda orientalis *were recognized in Iran (Figure 1).

A.crassicauda with *H.lepturus *and *O.doriae *have been implicated mostly of systemic envenomation. The envenomation syndrome observed in more severe cases are almost similar to that O.doriae except that is typically has more prominent neurotoxic manifestations (19). In general, stings from *A.crassicauda *result in severe and persistent pain occurring in majority of victims, enough severe to prevent the patient sleeping in almost all cases requiring specific intervention. It is imperative that victims have a high occurrence of ischemia around the wound, although a red appearance was confronted by the author. The similarity of clinical findings of *O.doriae *and *A.crassicauda *add further support to similar *in-vitro *observation of *O.doriae *(21) and *A.crassicauda *venoms in the isolated chick biventer cervicis nerve-muscle preparation (26). 

In a performed study on 30 people with *A.crassicauda *envenomation showed the observed manifestation are depend to the increased release of neurotransmitter by the cholinergic systems, specifically with the myocardial inotropic effect and increased pulmonary secretions. Severe pain is a prominent feature in admission envenomated by the second dangerous scorpion of Iran (26). Sudden death may be occurring during the first 24 hours due to cardiovascular arrest.

*A.crassicauda *shows high distribution in Azerbaijan, Turkey, Iraq, Syria, Jordan and Saudi Arabia as well (27, 28). The diverse toxicity of a species in different regions provides evidence for adaptive evolution and functional divergence of scorpion (29). Determination of clinical manifestations following *A.crassicauda *sting underling the venom have high neurotoxic components causes cholinergic system like poisoning with organ phosphor agents. As well as the majority of Iranian species, the available investigations about the venom of this species limited to clinical manifestations (14). Thus, there are no helpful studies in the characterization of the neurotoxic peptides and its biological activities to explain clinical manifestations and symptom severity. Recently, the cytotoxic and anti-cancer properties in SH-SY5Y and MCF-7 cells were addressed (30). However, recently four toxins from the venom of Turkey *A.crassicauda *were presented*. *Turkey specie having a Na(^+^)-channel α-type toxin peptide named Acra 4 (31-32). Acra3 administration induced severe neurotoxic events as seen similarly in clinical observations, such as: excitability and convulsions (33). 

The most records of the 23 scorpionism cases caused by Iranian *A.crassicauda *were in general siallora, nausea, vomiting, thirst, restlessness and increased of lung secretions. 

*H. lepturus: Taxonomy, envenomation and distribution*

The size of this type reaches 8 cm in males and 5.5 cm in females. Their body is yellow to light brown. They are unique because of their hermaphrodite looks point of view. They were found in 6 southern provinces at the height of 1000 m in maximum. Their distribution in the eastern part of Khuzestan province is higher.

This yellow scorpion is responsible for referral ranged from 10% to 15% for all referred cases of stings in Khuzestan province (15). The low prevalence of *H. lepturus *stings with severe symptoms suggest that its venom poses a significant risk to the health (34). Jalali and colleagues showed that, unlike *M. eupeus*, the intensity and severity toxic manifestations are associated with increased serum TNF-α levels and correlate positively with the clinical severity of the symptoms (35). The recorded data from clinical records of 354 hospitalized cases shows the local and systemic effect simultaneously (15). Large number of delayed and systemic disturbances, such as renal failure, hemolysis, in envenomed patients may be attributable to enzymatic components (36). The general symptoms more often reported were dry mouth, thirst, dizziness, nausea, fever and vomiting, and other symptoms associated with stimulation of sympathetic and parasympathetic systems. Ameliorating victims with fever, confusion, convulsion, hemoglubinuria and hemoglobin level below 10 g/dl from the severe toxicity has become a major concern in *H. lepturus *envenoming. Systemic and local reactions following sting, are usually local erythema, disseminated intravascular coagulation, renal failure and severe pulmonary hemorrhage (37, 38). At admission, almost the high percentage of victims shows the renal toxicity at the time of late referral to medical attention. The data showed that patients who developed renal changes were significantly more likely to have leukocytosis, reduction in urinary specific gravity, increased ESR, thrombocytopenia, hypoglycemia and coagulopathy with uncontrollable hemorrhage (15, 39). 

The toxicity arising from *H. lepturus *venoms differs significantly in both severity and based on the both in human and animal studies available in the literature (14, 19, 40-41). In the majority of recorded cases for *H. lepturus *envenomation, necrosis, ulceration of the skin, haemolysis of blood cells, and CNS (central nervous system) symptoms were associated with a poor outcome (11, 19). The CNS manifestations are seen mainly among children victims. The central toxicity effect decrease among the envenomated adults which implying the direct action of neurotic toxins on the central nervous system of infants not fully resistant and are likely the result of the venom’s peripheral action in adults (42). Recently, three toxins are isolated and characterized as described previously (11-13). 

However, the proper treatment of *H.lepturus *envenoming remains controversial, administration of available polyvalent anti-venom is only available treatment has not generally been agreed as the standard treatment protocol among the attending physicians for management of envenomated victims (41). The patients who received antivenin early during the envenomation had mild cardiovascular (43) and delayed renal manifestations*. *

While the antivenom is effective *in-vivo *and *in-vitro *(44, 45) severe clinical resulted following its stings that occur often and almost only in Khuzestan province. 

Clinical studies of the effects of the sting have been mainly carried out by Three reasearcher groups headed by Radmanesh and Pipelzadeh and recently Jalali (all from Jundishapur University of Ahvaz, Khuzestan) (4, 14-15, 19, 41). The clinical symptoms of *H. lepturus *stings cases vary considerably across recorded scorpion envenoming. The envenoming with renal, hepatic haematological disorders (19) and skin manifestations (15, 41) has made possible the report of different pattern in which the main clinical signs differ significantly. 

*Mesobuthus eupeus: Taxonomy, envenomation and distribution*

This scorpion reaches 5cm when is an adult. The color of its body is from light to dark yellow. There are dark spots at their back (middle parts) of this specious which are sometimes seen as the tidy and organized lines and in some of this kind which becomes a dark part at the back of the animal. This kind was hunted from the lowest part of the Khuzestan Province (Shadegan at the height of 5 m) to the highest point of the Zagros Mountains. Thus, this scorpion has both horizontally and vertically distributed in the regions. These yellow scorpions with different sizes widespread in the most parts of Iran and also found in neighbors countries (46). Five species, *M. gabrielis*, *M. agnetis*, *M. zarudnv*i, *M.eupeus *and *M.caucasicus *all belonging to the *Mesobuthus *genus were collected and recorded. 

*M.eupeus *is smaller than *A.crassicauda *species and characterized by the black or brownish spots look like stripe from external view, which are considered diagnostic feature for this scorpion so called spotted scorpion. It is recorded in many localities, desert as well as moderate climate of Iran.

In general, sting from *M.eupeus *result in minor local symptoms not requiring any specific intervention. However, it is considered a dangerous scorpion. In Iran*, *two species, *M.eupeus *and *M.caucasicus*, have been implicated in a small number of cases of systemic envenomation in humans. Indeed the most number of reported envenomations appears to be from southwest province, Khuzestan. The envenomation syndrome observed in more cases is not similar and severe to that of other dangerous species. When significant systemic envenomation occurs, the most symptoms which can reveal are neurogenic signs such as fever, vomiting, drowsiness, dyspnoea, hypertension, and tachycardia. These symptoms are fully recovered after administration of available antivenom. 

*Buthotus Saulcyi*: the size of this kind reaches 12 cm as an adult. Their color is from light yellow to dark yellow. The toxic gland (telson), which is at the end of the tail, has a black head and is one of the main parts of this specious. This kind was hunted from 6 South-Western provinces and has more spreading population compare to the other two ones. the highest part where these scorpions were caught, were at the «Hashtaad Pahloo mountain» from Lorestan Province with the height of 2300 m and the lowest area was at 300 m height in Khuzestan province (around Hoseiniye alliyya) (Table1; Figure 2).

*Non-medical important Iranian scorpions: Taxonomy, distribution*

According to the geographical location and different weather in the two specified regions, 40 areas from 10 southern provinces were under research for the scorpion hunting (Figure 2). Their characteristics and geographical spreading are all explained in the following part of this article. These samples are all kept in Razi Association for the toxic animal researches, so they can be seen by the visitors.

*Buthotus sach:*This animal reaches 13 cm as an adult. It has a dark brown or black color. Its body, specially its tail has too many villosity and that›s why this specious is known as the «Fuzzy» Black Scorpion (Pile Black Scorpion) .These kinds are from three south-western provinces and were hunted from the height of 500m to 1500m. There abundance is in concern in the MAHOUR MILATI area which is located at the South-Western province (Fars province) (Table1; Figure 2).

*Buthotus Jayakari*: This kind reaches 13 cm as an adult and has a light yellow to dark yellow color. The toxic gland, the last three cords of the tail, hands, jaws and the head are black which is one of the most descriptions of this kind. Its body, specially the tail and telson, has too many piles. This kind was hunted only in North-west of Fars province at the height of 1600-1800 m. This kind is mostly spread around horizontally in a limited way and is less likely to be seen in its living place (Table1; Figure 2).

*Compsobuthus matthiesseni*: The size of this kind reaches 4.5 cm as an adult. Its color is mostly from light to dark yellow. The tail cords are mostly thin and long, especially in the male ones. This kind was found in 4 south-western provinces. This study adds this dangerous scorpion to the major stinging scorpions in Khuzestan (47). They live in 300-1000 m high regions and are less likely to be found at the areas higher than a one kilometer. They have less population in their distributed areas (Table 1; Figure 2).

*Odontobuthus odonturus*: The size of this kind reaches about 8.5 cm when is an adult. The color of this kind is from light to dark yellow and is one of the excavator scorpions. This type is from the plain fields with the height of 50-1000 m of three South-west provinces were hunted. The distribution of this kind is not much at the studied region (Table1; Figure 2).

*Orthochirus scrobiculosus*: The size of this kind becomes 3 cm as an adult. The color of this kind is black but has a dark yellow hands and feet, which become lighter at their ends. The tail cords become thicker towards the back and there are few curved line was seen, especially on the 4^th^ and 5^th^ cords of the tail. This scorpion was hunted in 4 provinces from the height of 300 to 1000 m. Its distribution is limited in the studied region (Table 1; Figure 2).

*Razianus zarudnyi: *This scorpion reaches 3cm as an adult, and has a light yellow color. It has two side eyes at the front-side part of the head, which is one of specific parts of this kind. It was hunted from Fars and Khuzestan Provinces at 1000 m. This kind has a limit population in the geographical distribution (Table 1; Figure 2).

*Sassanidotus zarundnyi*: This species reaches 5cm as an adult and has a yellow color. There were found in Hormozgan Province and are less likely to be found at their homes (Table 1; Figure 2).

*Simonoides Farzanpayi*: The size of this scorpion reaches 3cm when is an adult and it has a black body. Its nippers, feet and shoulders are from brown to yellow, which become lighter towards the end. The cords of the tail get thicker towards the end, with curved lines especially on the 4^th^ and 5^th^ cords. The tail has loads of piles which is a specially feature of this kind. One of these types was found in Fars Province, in the height of Jahrom City. Their geographical distribution was the most limited one and there are so less likely to be at their living places (Table 1; Figure 2).

*Scorpio maurus*: The size of this kind reaches 7cm when is an adult and has a dark yellow to brown color. This scorpion is an excavator and was hunted in 3 provinces. They were found in the plain and warm places from 10 to 1000 m high. There are less likely to be found in their geographical distribution areas. This specie is belonging to scorpionidae family (Table 1; Figure 2).

*Habibiella persicus*: The size of this kind reaches 10cm when is a male adult and 7.3 cm when is a female adult and it has a dark yellow body. This type has hermaphrodite looks point of view as well. From this type there were only one hunted from Hormozgan Province (around the city of Bandar Abbas). This type as the Simonoides type has a limited distribution and was less likely to be found. This specie is belonging to scorpionidae family (Table1; Figure 2). 

## Discussion

Scorpion stings have been reported as a risk factor for Iranian health problems in most epidemiological studies over past decade. The present review indicates that a variety of stings, including fatal cases is associated with different varieties and types of scorpions in Iran, especially at the southern part. The southern parts of Iran are semi-warm areas and have the high scorpion populations. 

Iran has loads of different climate but is located at the moderate side close to equator and has too many plain fields and mountains according to the geographical situations. This country is bordered to Armenia, Azerbaijan and Turkmenistan on the north, to Persian Gulf and the Gulf of Oman on the south, To Iraq on the west, Turkey on the northwest and Afghanistan and Pakistan on the east. With all these, our information is so limited and less compare to what we should have achieved by now. One of the main reasons is that scorpions collected by Razi institute weren’t presented world-wide correctly. Iranian scorpion fauna remains one of the less well studied fauns. Details of the Iranian fauna and collection of species in Razi were not described sufficiently elsewhere. The results of yield and lethality of venom obtained from recognized scorpions collected in Razi during the past only summarized and presented by Latifi and Tabatabai in 1971 (7). Furthermore, the majority of hunting’s were done by the amateur people that didn’t have the enough knowledge of sample, collecting and hunting rules. In this study some notes are addressed about this remarkable diversity and high level of endemic species. Iran contains 17 genuses, 30 species and 18 sub-species that is more among Middle Eastern countries. Furthermore, the patterns of distribution of the fauna are commented and their geographical distribution is also enlarged. So it was considered that it is necessary to scorpion fauna of Iran well organized and studied in geographical distribution belief especially in the most populated regions of our country, about all the current information we have.

According to the existed results on geographical distribution pattern and the hunted types from the two broad southern regions, the high population, distribution and different types of scorpion in these regions of Iran were approved. Of these, *Androctonus crassicauda*, *Mesobothus eupeus*, *Buthotus Saulcyi *and *Hemiscorpius lepturus *had vast horizontal distribution, high population and density. The *Buthotus sach*, *Odontobuthus odonturus*, *Matthiesseni compsobuthus*, *Scorpio maurus *and *Scrobiculosus orthochirus *types had higher distribution and population after the previous mentioned ones. The *Razianus zarundnyi *(Fars and Khuzestan provinces), *Buthotus jayakari *(Fars), and *Sasaanidotus zarudnyi *(Hormozgan) ones are so limited in their distribution and they are less in population in their living regions.

The limited types are the *Simonoides farzanpayi *and *Habibiella persicus *ones. There were only one found from the first species close to Jahrom (Fars province) and one from the second species in Hormozgan Province. The most important achieved result was the different types and the abundance of Iranian scorpion fauna was more in the south-west compare to the south-east of the country. This could be connected to the earth tissues/textures, differences in the stones and plants of the regions, different in climate, different geographical situations, their nourishments and *etc*. Most of the areas (about 30) which considered for the scorpion hunting are located in south-west of Iran, in the provinces such as, Western Fars, Bushehr, Khuzestan, Ilam, Southern Lorestan and Bakhtaran.

The *Androctonus *and *Mesobuthus *types are in concern, as they are found in different climate, living circumstances in the provinces at the side of the Persian Gulf and Oman Sea, and the southern Zagros Mountain. These two were found in the warm and plain sites of the Persian Gulf and the Oman Sea, from the height of 5m to the high mountains of southern Zagros with the height of 2000 m. So the obtained results in this show that different mentioned species could resist different climate from the cold, moderate to the warm weather in a good way. The *Buthotus *types (*Jayakari*, *Shach*, *and Saulcyi) *were hunted from the high places of the Zagros Mountains with the height of more than 2000m to the height of southern provinces with the height of 300m. Even some of the samples of these types were collected from the high places, with the distance of few meters in the remaining snow, at the beginning of the spring. Therefore, they could be so strong in the cold weather. 

The *Odontobuthus odonturus *and *Scorpio maurus *scorpions are the excavator types and they dig their homes in the plain fields around the sea and in the plain fields of the Zagros regions and they have adopted themselves to the warm weather of the region. The other types, such as, *Hemiscorpius lepturus*, *Compsobuthus mattiesseni*, *Scrobiculosus orthochirus*, *Razianus zarudnyi*, *Simonoides farzanpayi*, *Sasanidotus zarudnyi *and *Habibiella Persicus*, are mostly found in the regions with the lowest height to 1000 m high areas. Thus, the warm and the moderate weather is so relevant and easy-going condition for these types.

All the scorpions found by the foreign and domestic researchers, are in two different families. There are Buthidae and Scorpionidae and are in seventeen different kinds. In all of these types, 15 of them are in southern Iran. *Apistobuthus*, *Butacus *and Palpater are the ones which haven’t been under research in this study. Even in the past thirty five years, which loads of scorpions were given to the Razi association for the toxicology and zoology; there were no more than few of these kinds found and hunted. Therefore, it seems like that recent types had a limited distribution in Iran and weren’t found more often.

However, with all these geographical distributions, 14 types of the Iranian scorpions were under the research and study, and there were thirty institutes for hunting, created for the dangerous Iranian scorpions.

The important thing is that, about 5 of the Iranian scorpions which are in the list of the most dangerous scorpions in the world (4) have had a vast distribution and have a high population in the southern regions of our country. Therefore, with teaching and guiding the hunters and sending them towards the mentioned institutes, we can hunt enough number of dangerous scorpions. So by seeking to get the toxin/venom of the scorpions, we can improve our antivenin and serum knowledge and achievements against the scorpion stings and step forward in a serious and successful way.

*Conclusions, future directions, and further applications*

Despite a recent upsurge of interest in scorpion venoms by various research groups, there remain many challenges for Iranian toxinologists. Clinically, there exists the unresolved issue of the effectiveness of available antivenom, the clear clinical origin of *H.lepturus *scorpionism and often the unknown mechanism of action and severity reason of envenomation in several scorpion species of Iranian scorpions.

Given the little number of investigated Iranian scorpion venoms, there exists a vast treasure of not analyzed and isolated toxins. A few of these scorpion toxins are already being investigated as molecular tools in the pharmacology and electrophysiology to define the subtype and structure–function of mammalian and/or insect ion channels.
